# Complete genome sequence of *Fusobacterium vincentii* strain TDC100 isolated from an apical periodontitis lesion

**DOI:** 10.1128/mra.01197-23

**Published:** 2024-02-09

**Authors:** Hideo Yonezawa, Eitoyo Kokubu, Yuichiro Kikuchi, Kazuyuki Ishihara

**Affiliations:** 1 Department of Microbiology, Tokyo Dental College, Tokyo, Japan; Rochester Institute of Technology, USA

**Keywords:** *Fusobacterium vincentii*, complete genome, apical periodontitis

## Abstract

This study reports the complete genome sequence of *Fusobacterium vincentii* strain TDC100. The complete circular chromosome of strain TDC100 was obtained and assembled using a combination of short- and long-read sequencing.

## ANNOUNCEMENT

*Fusobacterium vincentii* is a Gram-negative, obligate anaerobic bacterium belonging to the family Fusobacteriaceae (1). This bacterium, formerly known as *Fusobacterium nucleatum* subsp. *vincentii*, was reclassified as *F. vincentii* in 2021 (2, 3). *F. vincentii* is most abundant in the human oral cavity, particularly in dental plaques, and acts as a bridge bacterium that facilitates colonization by other bacteria via aggregation (4). Here, we report the complete genome sequence of *F. vincentii* strain TDC100, which was isolated from an apical periodontitis lesion in 2008 at Tokyo Dental College Chiba Hospital (5). This strain exhibits strong adherence to type-I collagen and biofilm formation (5–8).

**Fig 1:**
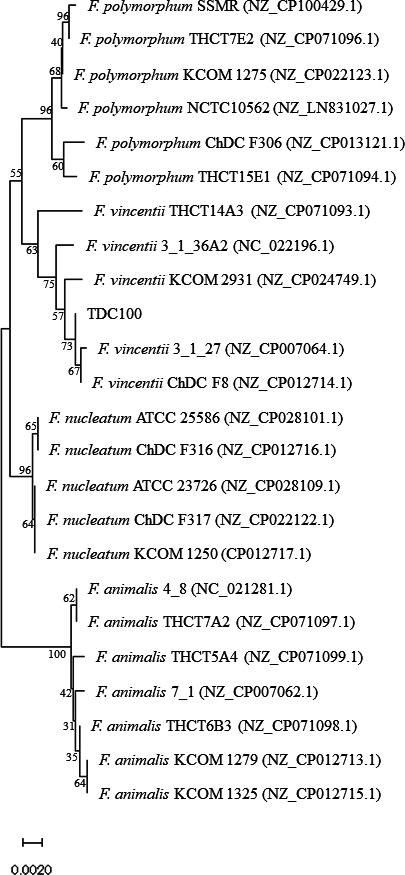
Phylogenetic analysis of strain TDC100 based on 16S rRNA gene sequences. The phylogenetic tree was generated via the neighbor-joining method using the MEGA11 software with MUSCLE (version 1,0,13) based on default parameters (14). Genetic evolutionary distance model was computed using the Tamura-Nei method, and the percentage reliability values for each node were determined by bootstrap analysis with 1,000 replicates. GenBank accession numbers are provided in parenthesis. Scale bar indicates nucleotide substitutions per site.

The strain TDC100 was stored at −80°C in Tryptic soy broth containing 20% glycerol. The cells were maintained and cultured on Gifu Anaerobic Medium (GAM) agar (Nissui, Tokyo, Japan) under anaerobic conditions. A single colony was cultured in GAM broth at 37°C overnight under anaerobic conditions. Genomic DNA was extracted using the Quick-DNA Miniprep kit (ZYMO RESEARCH, Irvine, CA). The 1 µg of genomic DNA was treated with Short Read Eliminator XS (Circulomics Inc. Baltimore, MD) to remove low-molecular-weight DNA of approximately 10 kb or less. The 600 ng of genomic DNA was prepared using a ligation sequencing kit (SQK-LSK109) and sequenced on GridION platform (Oxford Nanopore) using FLO-MIN106 R9.4.1revD. The remaining 400 ng of DNA was prepared using an MGIEasy FS DNA Library Prep Set (MGI Tech Co., Ltd.) and sequenced using DNBSEQ G400RS (MGI). The runs generated 5,285,473 read pairs with 792,820,950 bases of short reads, and 273,387 reads with 1,545,963,065 bases of long reads. The average Nanopore read length and N50 were 5,655 bp (range 101–107,194 bp) and 9,172 bp, respectively. The adaptor and barcode sequences and low-quality reads (average Phred quality value of 30.0 or 10.0, and short reads of 20 bp or 3,000 bp for short-read sequencing or long-read sequencing, respectively) were removed using Fastp (ver. 0.20.1) (9) or NanoFilt (ver. 2.7.1) (10). The short and long reads had a mean coverage of 300× and 160×, respectively. Default parameters were used for all software unless otherwise specified. Hybrid-assembly was performed using Unicycler (ver. 0.4.8) (11), and the quality-filtered reads generated a circularized chromosome and rotating assemblies to begin at a consistent starting gene (*dnaA*), which was determined based on the Bandage plots from the Unicycler output. The whole-genome sequence comprises 2,075,724 bp with a GC content of 27.2%. Phylogenic analysis using 16S rRNA gene divides *Fusobacterium* into four species (12). The 16S rRNA gene phylogenic analysis showed that the strain TDC100 was grouped into the cluster of *F. vincentii* (Fig. 1). *F. vincentii* contains two subspecies, previously named *F. nucleatum* subsp. *vincentii* and *F. nucleatum* subsp. *fusiforme* (1); however, the classification below the species level requires more careful evaluation of the phenotype. Therefore, we present the classification results only down to the species level. The genome sequence was annotated using DFAST (ver. 1.6.0) (https://dfast.nig.ac.jp) (13).

## Data Availability

The genome sequence was deposited in NCBI/ENA/DDBJ under the accession number AP028933. The sequencing data are available in the DDBJ Sequence Read ARCHIVE under the accession numbers DRX497976 and DRX497977.
